# Supplementary material for: Mental models use common neural spatial structure for spatial and abstract content
Source: Commun Biol. 2020 Jan 9;3:17. doi: 10.1038/s42003-019-0740-8 (PMC6952387; doi:10.1038/s42003-019-0740-8)
Supplement: Supplementary file 1 — Description of Additional Supplementary Items [file 42003_2019_740_MOESM1_ESM.pdf]

Description of Additional Supplementary Files

File Name: **Supplementary Data 1**

Description: This Excel formatted file contains the source data for the two graphs displayed in Figure 1 (graphed with ggplot2 in R). The first tab contains the Subject ID, Session #, Content Type, and Accuracy for the Paired Heights task. The second tab contains the Subject ID, Session #, Content Type, and Spearman Rho correlation between the participant-reported ordering for each content problem space and the correct ordering. Each of these tabs are sorted with ascending Subject ID, then Content Type, followed by Session #.
