# Supplementary material for: Mental models use common neural spatial structure for spatial and abstract content
Source: Commun Biol. 2020 Jan 9;3:17. doi: 10.1038/s42003-019-0740-8 (PMC6952387; doi:10.1038/s42003-019-0740-8)
Supplement: Supplementary file 3 — Reporting Summary [file 42003_2019_740_MOESM3_ESM.pdf]

nature research

Corresponding author(s): Katherine L Alfred

Last updated by author(s): Nov 20, 2019

Reporting Summary

Nature Research wishes to improve the reproducibility of the work that we publish. This form provides structure for consistency and transparency in reporting. For further information on Nature Research policies, see Authors & Referees and the Editorial Policy Checklist.

Statistics

For all statistical analyses, confirm that the following items are present in the figure legend, table legend, main text, or Methods section.

n/a	Confirmed	
☐	☒	The exact sample size (*n*) for each experimental group/condition, given as a discrete number and unit of measurement
☐	☒	A statement on whether measurements were taken from distinct samples or whether the same sample was measured repeatedly
☐	☒	The statistical test(s) used AND whether they are one- or two-sided<br>*Only common tests should be described solely by name; describe more complex techniques in the Methods section.*
☐	☒	A description of all covariates tested
☐	☒	A description of any assumptions or corrections, such as tests of normality and adjustment for multiple comparisons
☐	☒	A full description of the statistical parameters including central tendency (e.g. means) or other basic estimates (e.g. regression coefficient) AND variation (e.g. standard deviation) or associated estimates of uncertainty (e.g. confidence intervals)
☐	☒	For null hypothesis testing, the test statistic (e.g. *F*, *t*, *r*) with confidence intervals, effect sizes, degrees of freedom and *P* value noted<br>*Give P values as exact values whenever suitable.*
☒	☐	For Bayesian analysis, information on the choice of priors and Markov chain Monte Carlo settings
☐	☒	For hierarchical and complex designs, identification of the appropriate level for tests and full reporting of outcomes
☐	☒	Estimates of effect sizes (e.g. Cohen's *d*, Pearson's *r*), indicating how they were calculated

Our web collection on statistics for biologists contains articles on many of the points above.

Software and code

Policy information about availability of computer code

Data collection	A PsychoPy-based (version 3.0) python script was generated for data collection alongside the data from the functional MRI. The script was minimally modified to include scan trigger inputs to ensure the script and the MRI remained in sync.
Data analysis	FSL (version 5.0) was used to register and motion correct the data (MCFLIRT) and obtain beta values from the GLM model (FEAT). Data was prepared for surface analysis using Freesurfer recon-all. RSA analyses were conducted using the PyMVPA (2.7) suite (pdist).

For manuscripts utilizing custom algorithms or software that are central to the research but not yet described in published literature, software must be made available to editors/reviewers. We strongly encourage code deposition in a community repository (e.g. GitHub). See the Nature Research guidelines for submitting code & software for further information.

Data

Policy information about availability of data

All manuscripts must include a data availability statement. This statement should provide the following information, where applicable:

- Accession codes, unique identifiers, or web links for publicly available datasets
- A list of figures that have associated raw data
- A description of any restrictions on data availability

Data and code will be made available upon request.

Field-specific reporting

Please select the one below that is the best fit for your research. If you are not sure, read the appropriate sections before making your selection.

☐ Life sciences ☒ Behavioural & social sciences ☐ Ecological, evolutionary & environmental sciences

Behavioural & social sciences study design

All studies must disclose on these points even when the disclosure is negative.

Study description	This is a within-subject representational similarity analysis study, comparing the pattern of neural activity while participants are reasoning about the relative positions of items in a mental model created through transitive reasoning with an a priori model of the hypothesized structure of that mental model.
Research sample	Dartmouth undergraduate students (19 total, 15 female; mean age= 19.81) who were fluent in English and right-handed participated in this study. No participants had a history of neurological or psychiatric disorders.<br><br>The sample was chosen as a convenience sample of adults. There are currently no hypotheses predicting differences in the transitive reasoning process between adults with no neurological problems, allowing us to use any sampling of adults with no history of neurological or psychiatric disorders. Sample size is based on our previous study and others with similar effect sizes.
Sampling strategy	Participants enrolled in this study through the Dartmouth online SONA system for recruitment for psychological and brain science studies. Participants were recruited if they met the basic requirements (minimum age of 18, right hand dominant, and fluent English speaker). Sample size is based on our previous study and others with similar effect sizes.
Data collection	Data collection was collected via pen and paper for the Hierarchy Reconstruction task, and was collected via computer for all other tasks. No other people were present during data collection other than the participant and the researcher.
Timing	May 2016-June 2016
Data exclusions	No data excluded
Non-participation	One participant withdrew from the study after behavioral sessions but at the beginning of fMRI data collection because that participant felt the protective ear wear was too uncomfortable to continue the scan. That participant's incomplete dataset was not included in any analyses.
Randomization	Participants were not sorted into groups using any method. All conditions were within-subject in one group.

Reporting for specific materials, systems and methods

We require information from authors about some types of materials, experimental systems and methods used in many studies. Here, indicate whether each material, system or method listed is relevant to your study. If you are not sure if a list item applies to your research, read the appropriate section before selecting a response.

Materials & experimental systems

n/a	Involved in the study	
☒	☐	Antibodies
☒	☐	Eukaryotic cell lines
☒	☐	Palaeontology
☒	☐	Animals and other organisms
☐	☒	Human research participants
☒	☐	Clinical data

Methods

n/a	Involved in the study	
☒	☐	ChIP-seq
☒	☐	Flow cytometry
☐	☒	MRI-based neuroimaging

Human research participants

Policy information about studies involving human research participants

Population characteristics	See above
Recruitment	Participants selected and enrolled in the study through the Dartmouth SONA online scheduling system.
Ethics oversight	Dartmouth's Committee for the Protection of Human Subjects approved this study (under protocol 23887).

Note that full information on the approval of the study protocol must also be provided in the manuscript.

Magnetic resonance imaging

Experimental design

Design type | A block-related design with variable-length fixation durations between stimuli were used.

Design specifications | There were a total of 6 functional runs (2 runs per content type by 3 content types) per participant. Each of the runs included 148 volumes per run for 888 volumes total. Between each trial, there was a variable-duration fixation cross (between 5-20s) and trials varied between 15 and 30s in duration (5s for each of two simuli, plus a second 5-20s variable fixation cross in between).

Behavioral performance measures | Response time and accuracy was collected for all participants. Because the task design that would provide the best isolated signal for that particular item included a relatively subjective assessment of general location in the item space, we collected data from two other tasks during that same session that would allow us to better quantify participant knowledge of the problem space. Further, participants were trained during two prior behavioral sessions to ensure that participants fully understood the task they would be performing in the scanner.

Acquisition

Imaging type(s) | Functional

Field strength | 3T

Sequence & imaging parameters | For the functional runs, there were six (2[run]x[3 content type]) runs of 148 volumes per run for a total of 888 functional (T2*) volumes with a TR of 2.5s. The functional scans were collected using gradient-echo EPI with 42 Philips interleaved transverse slices at 3 mm per slice (TE = 35, flip angle = 90 degrees).

Area of acquisition | The whole brain was included in the area of acquisition for all scans.

Diffusion MRI ☐ Used ☒ Not used

Preprocessing

Preprocessing software | Data were pre-processed using FSL's MCFLIRT script to register the functional data to each subject's anatomical image as well as MNI 2mm standard space. Brain extraction and spatial smoothing (5 mm FWHM Gaussian kernel) were performed at this stage, and data were checked to ensure no more than 4mm of head movement.

Normalization | All data were registered to the standard 2mm MNI brain.

Normalization template | Transformation to the standard 2mm MNI brain was calculated using FSL's MCFLIRT using 12 degrees of freedom.

Noise and artifact removal | No artifact removal was performed

Volume censoring | There was no volume censoring

Statistical modeling & inference

Model type and settings | The following analyses were performed using Python and PyMVPA (http://www.pymvpa.org; Hanke et al., 2009), SciPy (http://scipiy.org), and NumPy (http://numpy.scipy.org). The searchlight-based representational similarity analysis (RSA) was conducted on the neural surface, using a 100 voxel searchlight mapping technique (Oosterhof et al., 2011) that produced a whole-brain map on the group level (created using the average of the individual subjects untargeted neural similarity for each content condition) that reflected the Pearson correlation between local neural representational structure and a target similarity structure for each of the content conditions (Height, Price, and Abstract; Figure 2). Each modeled dissimilarity matrix was created using the ordinal ranking of the objects, where the tallest/most expensive/"vilchiest" object is one distance away from the second object, two from the third object, and so on for all five items in each hierarchy. At each searchlight location, the local neural dissimilarity matrix was computed using correlation distance between activity patterns (derived from beta values in the item-level GLM, described above) for all pairs of stimuli within that content type. Activity patterns were defined by the voxel-wise estimated hemodynamic responses from GLM analysis of the functional data collected during the two Hierarchy Probe runs for each of the three content conditions (excluding the portion of the trial in which a button response occurred). After all individual untargeted neural dissimilarity matrices were calculated, a group average was calculated within each content type. Each of these content-specific average neural maps were then correlated with the modeled dissimilarity matrices for item rankings within each content type. The resulting correlations were permutation-corrected against a null distribution of 10,000 randomized potential correlation maps, resulting in a corrected Z-map for each content type (Figure 3) indicating the likelihood that any observed correlation was significantly different from chance. For further details, please see the section on multiple comparison correction below.
These three content-specific maps were further used as input for the higher-level Rank Average RSA analysis. In this analysis, we aimed to identify neural regions that showed patterns of activity that matched the predicted structure for the mental models across multiple content types. We chose to average the content-specific permutation-corrected z-maps rather than find the conjunction of all three content-specific maps because we were concerned about the potential rate of Type II error coming from only considering results that had passed several levels of strict corrections. Through our generated distributions from our permutation corrections (described in detail below), we identified that

the noise threshold was z > 1.37. Given we were testing our hypothesis about a positive correlation between the theoretical mental model and the pattern of neural activity, we set our cutoff for the threshold for minimum values from each of the permutation-corrected content-specific maps as z > 1.65, a one-tailed p < .05. In short, when the average rank z-map was calculated, only permutation-corrected z values greater than 1.65 from each of the content-specific RSAs were included. All other values were set to 0 to prevent averages being artificially. The content-specific permutation-corrected z-maps that were thresholded at z > 1.65 were averaged together to create one average map that represented relative rank of items, across content types. We then used AFNI's surface-based cluster simulation to identify significant clusters on the surface (clusters would be required to have an area greater than 120 mm2, at a bootstrap-corrected threshold of p < .05, corrected).
After clusters were identified, we further verified that the clusters were not due to an exceptionally high value from a single content specific map. To do so, we identified that the maximum permutation-corrected z that was included in the average was 4.1 Given the average map was calculated from three maps, an average between 4.1 and two additional 0s (non-significant values from the other two maps were masked as 0) resulted in a z of 1.36. This maximum value is the highest possible value that could have resulted from a single map contributing to a region in the average map. The cluster in the right IPS, left IFC, and the left aPFC both had peak and cluster-average z values higher than that cutoff, indicating that both of those clusters could not have possibly been present due to the results from a single content-specific map, and minimally indicate that multiple types of content show the same pattern of activity in those regions.
Finally, to investigate which regions in the average RSA analysis represented similar mental models in each of the three content types, we calculated a conjunction map. Each of the original content-specific RSA maps were thresholded z > 1.65, p < .05 (as with the average map above). Then, each content-specific map was binarized, so that each node greater than the minimum threshold had a value of 1 and all other nodes were 0. The three binarized maps were then multiplied by different values and summed together. Different values were assigned to each map so it would be clear which content types were overlapping in which regions (which would be lost through simply binarizing the maps and summing).

Effect(s) tested

The whole-brain searchlight RSA identified regions of the brain where the pattern of neural responses to a participant considering the placement of a given item in a space significantly correlated with the predicted pattern of dissimilarity between the items in the problem space, given correct transitive inference.

In the case of the average-content RSA, the analysis would identify the regions that are common between each of the content-specific whole brain searchlight RSA analyses. This allows us to identify regions that represent the common structure of transitive reasoning models across a variety of models that ranged from fully concrete to fully abstract. The conjunction analysis further allowed us to determine which content types were overlapping in each given region revealed by the average RSA.

Specify type of analysis: ☐ Whole brain ☐ ROI-based ☒ Both

Anatomical location(s)

To support our assertion that the involvement of the right IPS represents spatial information specifically, we used the association map from NeuroSynth with the "spatial" keyword (NeuroSynth.org; Yarkoni et al., 2011). This map indicates areas that are selectively active for spatial information as compared to all other terms in the database (created through meta-analysis of 1,157 studies that include "spatial" as compared to the remaining 13,214 studies, thresholded at FDR corrected p < .01). This approach is an objective, external, data-driven method to generate networks based on keywords in which activity across previous studies has been associated more with the term "spatial" than with other terms in the database. The spatial association map was binarized and the clusterized average z-map of the permutation-corrected and cluster-corrected RSA was overlaid on top of the spatial association map.

Statistic type for inference
(See Eklund et al. 2016)

We used correlation (goodness of fit) between neural data (distance matrix) and a hypothesized model. The statistic type used for inference was the surface node-wise permutation-corrected z-scored likelihood of the actual r-values.

Correction

We conducted a permutation test to compare our predicted dissimilarity matrices and observed results to a distribution of possible results based on a distribution of 10,000 random permutations of the target labels. The probabilities associated with our results were thus calculated as the z-scored likelihood of the actual r-values occurring by chance at a given node compared to a distribution of possible outcomes created by shuffling the data 10,000 times. In order to examine the effects of rank across content types, we averaged the permutation-corrected z-maps for each content type, thresholded at z > 1.65, one-tailed p < .05. This average map for rank was further bootstrap cluster corrected using AFNI's 3dClustSim function for surface clusters significant at p < .05 (120 mm2).

Models & analysis

n/a	Involved in the study
☒	☐ Functional and/or effective connectivity
☒	☐ Graph analysis
☐	☒ Multivariate modeling or predictive analysis

Multivariate modeling and predictive analysis

The RSA model for each content type was created by using the rank ordering of items in the transitive problem space to create the dissimilarity matrix. To clarify, in a given content space, there were five items. Each item could be ranked in order from most to least in that dimension (e.g. the most expensive to least expensive). The item that was ranked first in a content space was assigned 1 distance to the second item in that space, 2 distance to the third item, and so forth. The models for each content RSA were modeled separately. The cross-content RSA did not have a separate RSA model to prevent confounding of examining similarity within a content domain while also analyzing content across domains.
